# Laparoendoscopic Single Site Hysterectomy: Literature Review and Procedure Description

**DOI:** 10.3390/jcm10102073

**Published:** 2021-05-12

**Authors:** Liliana Mereu, Francesca Dalprà, Saverio Tateo

**Affiliations:** Gynecological and Obstetrics Department, Santa Chiara Hospital, 38122 Trento, Italy; Francesca.dalpra@apss.tn.it (F.D.); Saverio.tateo@apss.tn.it (S.T.)

**Keywords:** LESS hysterectomy, single site laparoscopic hysterectomy, robotic-LESS, review

## Abstract

Laparoendoscopic single site surgery (LESS) refers to a spectrum of surgical techniques that allow the performance of laparoscopic surgery through consolidation of all ports into one surgical incision. LESS has emerged as a potentially less invasive alternative to multiport laparoscopy and in the last year in gynecology; hence, this approach has been largely applied for selective indications to perform total hysterectomy. We performed a literature review on single site hysterectomy and described indications and technique, highlighting practical problems, pointers, limitations and recent technical development as robotic assistance.

## 1. Rationale

Laparoendoscopic single site surgery (LESS) is a novel surgical approach that has been associated with reduced postoperative pain, improved cosmetic outcome, avoidance of ancillary port complications and faster recovery compared to multiport laparoscopy [1].

LESS is more technically challenging compared to standard laparoscopy and requires the coordination of multiple instruments through a single small incision. In recent years, the development of new technology and instrumentations such as robotic assistance has been developed to overcome ergonomic complexity and has permitted a diffusion of the single site approach. In the last few years, an increasing amount of literature has been published on single site laparoscopic hysterectomy for benign and malignant indications. The objective of this study is to highlight technique, tips and tricks, indications and outcomes of LESS hysterectomy through the revision of the most recent literature.

## 2. Introduction

Hysterectomy is the most common major gynecologic surgery performed worldwide. Hysterectomy can be performed abdominally, vaginally or laparoscopically. Laparoscopic approaches include total laparoscopic hysterectomy (TLH), laparoscopic-assisted vaginal hysterectomy, and robotic-assisted laparoscopic hysterectomy.

Minimally invasive surgery should be used whenever feasible, due to the well-known advantages of excellent cosmesis, shorter hospital stay, faster recovery and return to routine activities, decreased blood loss, decline in wound related complications such as infection, hematoma and dehiscence, and other major postoperative complications such as deep vein thrombosis and respiratory morbidities.

In the last few decades, there has been an increasing interest in even less invasive surgery such as mini-laparoscopy, LESS, and natural orifice endoscopic surgery (NOTES), and the development of new specific instruments has led to a gradual application of these new approaches in routine practice.

LESS surgery is an attempt at improving cosmetic outcome, faster recovery and decrease postoperative pain and hospital stay. Furthermore, reduction in the number of ports would also mean reduction in the port-associated complications like hernia, vascular and soft tissue and nerve injuries during trocar insertion.

## 3. LESS Hysterectomy

A specially designed single multichannel port provides access for the laparoscope as well as several other laparoscopic instruments. A variety of dedicated port systems and instruments are available for single incision laparoscopy. Port systems vary in number of channels and diameters, (3 to 5 channels, ranging from 3 to 15 mm) so determining the length of fascial incision needed. Most are disposable; however, reusable devices are also available, with the advantage of reducing cost.

Surgical instruments used in conventional single site TLH are: 30 degree 5 mm endoscope, laparoscopic 5 mm bipolar forceps, monopolar hook, scissors, needle holder, suction irrigation device and uterine manipulator. In order to improve performance of this surgical approach, specialized instruments and some ergonomic techniques have been improved.

Laparoscopic surgical techniques are based primarily on traction and counter traction forces that allow triangulation of forces applied from two different points. Inserting the laparoscopic instruments from a single-site port makes triangulation difficult for the deployment and visualization of the operating field when using standard laparoscopic in-line instruments. In this setting, a parallel alignment of the laparoscopic tools reduces the range of motion between them and it is often associated with counterintuitive movements on the outside, where the distal ends of standard laparoscopic instruments are prone to clash with each other. For this reason, it can be necessary to cross instruments during single site surgery and it may be useful to have a curved instrument that with rotation on its shaft axis or with a cranio-caudal movement, permits tissue traction, reducing clashing of the instruments.

A multifunctional versatile laparoscopic device with simultaneous grasping, coagulation and transecting function can help in reducing instruments exchange and consequently operating time. A 30° laparoscope permits a correct visualization of both sides of the pelvis by simple rotation on its axis preventing inadvertent contact with the operating instruments. Intra-uterine manipulator permits a better exposure of the pelvic field.

The patient is placed in dorsal lithotomic position with both arms close to the body with open legs and thighs flexed, allowing the assistant to handle the uterine manipulator. It is of key importance that the assistant can mobilize the uterus transvaginally, in order to help the surgeon by exposing the correct surgical planes of the operative field.

A 2–3 cm umbilical skin incision is performed with a pointed scalpel. The choice of transverse versus vertical incision depends upon anatomy of the umbilicus and the direction should be selected to minimize the incision length within the umbilicus to improve cosmesis and the incision should be kept as much within the umbilical ring as possible. With small retractors, the rectus fascia is identified and grasped with two Kocher clamps, the fascia is opened with a scalpel and the peritoneal opening is enlarged under direct vision with the surgeon’s finger or with the use of blunt scissors. Fascial suture can be placed at the fascia edges to facilitate closing. Once the peritoneal cavity has been entered, the single port device can be inserted and accurate inspection of the abdomen is performed with the endoscope.

**Figure 1:**
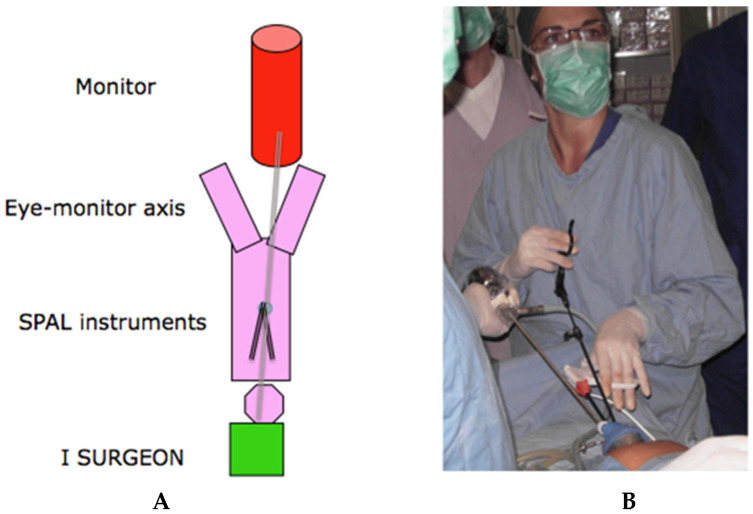
(**A**) Operative room ergonomy. (**B**) First surgeon ergonomy.

To provide an ergonomic eyes–hands–monitor axis, the first surgeon stands at the head of the patient using two (left and central) port accesses. The assistant stands at the level of the patients’ right shoulder holding the endoscope in the right port access and the monitor is placed between the patient’s legs (Figure 1A,B).

LESS total hysterectomy follows identical steps of multiport TLH [1]. Bilaterally, the ovarian ligament, fallopian tubes and round ligaments are coagulated and transected; the broad ligament is dissected anteriorly and inferiorly towards the bladder, and the bladder is carefully dissected off the uterus. Posterior, the broad ligament is dissected up to the uterosacral ligament at the level of uterine torus. Uterine vessels are skeletonized, coagulated and transected. Circumferentially, colpotomy is performed along the valve of the uterine manipulator. Different methods may be used to maintain pneumoperitoneum during colpotomy, generally strictly related to the type of uterine manipulator; 360-degree vaginal valve, sponge or silicone vaginal occluder. The uterus is removed through the vagina and, if too big, inside an endobag with cold knife morcellation. Vaginal cuff is closed using 2-0 V-Loc sutures in a continuous running fashion.

## 4. Robotic LESS Hysterectomy

A recent advantage in LESS hysterectomy has been the development of robotic single-site technology with an overcoming of the technical limitations of LESS.

Robotic technology improves vision, dexterity, precision motion scaling, tremor control, reduces deficiency of port triangulation, clashing instruments, single site confusion, ergonomic complexity and, consequently, learning curve. Moreover, the possibility to have an endoscope with high definition three-dimensional vision and fluorescence technology permits its use also in early stage endometrial and cervical cancers.

**Figure 2:**
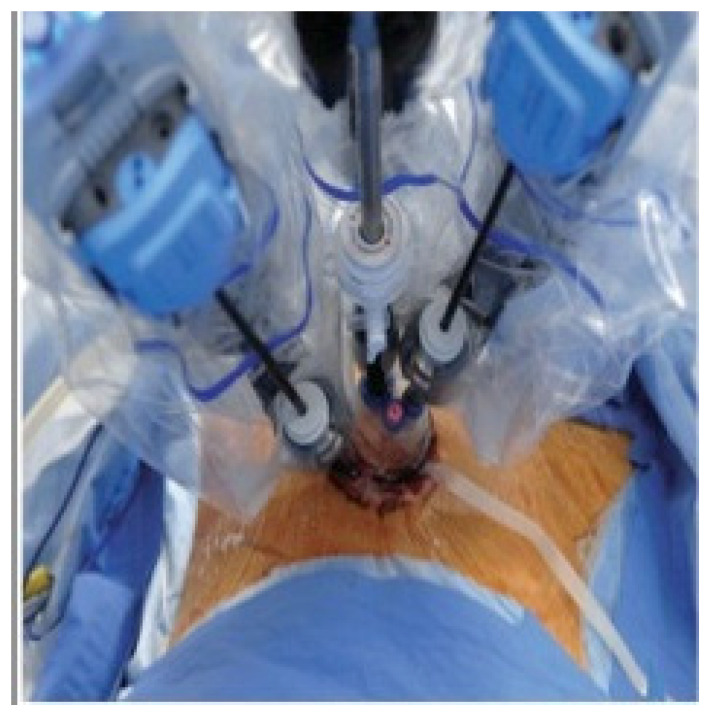
R-LESS set up.

The surgical technique follows the steps previously described [2]. The single port device is a multichannel disposable specific for the da Vinci Surgical system with space for four cannulae and an insufflation valve (Figure 2). The specific cannulae used are: two 5 mm × 250 mm-long curved cannulae for robotic flexible instruments, one cannula for 8 mm endoscope and one 5 or 10 mm laparoscopic cannulae for the bedside assistant surgeon.

After the patient has been placed in the lithotomic 30° Trendelemburg position, the Da Vinci Robot is docked and specific robotic bipolar and monopolar hooks are inserted while the assistant helps with suction irrigation or forceps.

At the end of the hysterectomy the surgical specimen is removed through the vagina and the vaginal cuff closure is performed intracorporeally with a 2-0 V-Loc suture in a continuous running fashion with robotic wristed needle holder.

## 5. Discussion

In 1991, Pelosi realized the first laparoscopic hysterectomy with single umbilical access [3]. However, it was not widely accepted by gynecologic surgeon due to technicalities. Single port laparoscopy has enjoyed resurgence thanks to recent technological advances in endoscopic instrumentations, specific port systems and optics [4,5,6].

Hysterectomy may require a higher level of surgical skill that adnexal surgery does; nevertheless, LESS is now widely applied for not only laparoscopic-assisted vaginal hysterectomy (LAVH) but also total laparoscopic hysterectomy (TLH) [5,6,7]. In 2015, a retrospective study reported that 80% of hysterectomies in a single hospital in Korea were performed via LESS [8].

Although there have been an increasing number of studies comparing the surgical outcomes of single port laparoscopic hysterectomy (LH) and conventional multiport LH, the results are conflicting and few are RCTs has been conducted (Table 1).

A recent metanalysis [17] of LESS and MPL hysterectomy evidences that when LESS and MPL were compared, there was a shorter OP time for MPL (SMD = −0.2577, *p* < 0.001) and lower rate of transfusion (OR = 0.1697, *p* < 0.001), without a significant difference in EBL (SMD = −0.0243, *p* = 0.689). There was a nonsignificant trend toward higher risk of conversion to laparotomy in the MPL group (OR = 2.5871, *p* = 0.078). Pain scores were no different 12 or 24 h postoperatively but were significantly higher at 48 h postoperatively (SMD = 0.1861, *p* = 0.035) in the MPL group. There were no differences in overall or individual complications between the LESS and MPL. The single-port technique for benign hysterectomy is feasible, safe, and equally effective compared to the conventional technique. No clinically relevant advantages were identified, and no data on cost effectiveness are available.

Tuoheti et al. [18] performed a metanalysis on LESS vs. MPL hysterectomy for endometrial cancer including four studies and 234 patients. No statistically significant difference in complications, blood loss, surgical time, hospital stay, number of lymph nodes. They evidenced that LESS had more pelvic lymph node removed and improves cosmesis.

The single port approach should be considered a regular laparoscopic procedure because its successful implementation involves a significant learning curve. Stepwise practical training on simulators, animal models, and under supervision of an experienced tutor is mandatory before performing this technique in operating theatre. The learning curve in achieving sufficient skill for single–port access hysterectomy even for well experienced multiport laparoscopists, could be reached after at least 10–15 and up to 40 surgeries [19,20].

In general, it must be taken into consideration that challenging situations such as large uteri, overweight patients and previous abdominal surgery can affect the outcome of our patients and should, therefore, be avoided during the learning curve. In the clinical routine, LESS hysterectomy may be consider for patients without large uterus and with a contraindication to vaginal approach such as adenomyosis, mild fibromatosis (<20 weeks), low risk and precancerous endometrial and cervical cancer, BRCA mutation risk reducing, sex change, LAVH with abdominal adhesions or with adnexal pathologies.

While robotic technology has improved surgical ergonomy in single site surgery, further developments are needed to improve this approach. Curved trocars in the robotic platform reduce the effective surgical space in the abdomen and pelvis; it can be more difficult to access all anatomy surrounding large uteri or specific spaces at the level of the umbilicus such as the promontorium. Another limitation is the rigidity of the system; the absence of wrist technology for the majority of the instruments reduces the benefit of robotic technology.

The complication rate can reach 4.9% while the conversion rate can reach 2.8%. Comparative studies did not reveal any difference in terms of intra and postoperative complications between R-LESS and LESS hysterectomy [21]. Robotic LESS has been shown to be safe and feasible for laparoscopic hysterectomy (Table 2).

Many clinicians have reported feasibility and safety of LESS also for oncological indications as endometrial and cervical cancer [40,41].

Although the surgical indications for MLS has slowly extended to more advance gynecologic pathologies, robotic minimal invasive surgery has been rapidly utilized in various gynecologic disease. Consequently, robotic laparoendoscopic single site (R-LESS) instruments have also been developed and R-LESS hysterectomy is becoming more standardized and is increasingly used by surgeon both for benign and malignant pathologies. A retrospective comparative study between R-LESS and LESS hysterectomy for benign indications in 100 patients found no significant difference in terms of conversion to multiport procedure and blood loss; however, the operative time was 24.9 min longer in the R-LESS [35]. The feasibility and safety of R-LESS hysterectomy +/− pelvic lymphadenectomy or sentinel lymph node detection for early endometrial cancer has been demonstrated by certain number of studies [21,22,33,42].

In terms of learning curve, the most difficult procedure is vaginal cuff suture and proficiency can be achieved after 14 cases. In general, large uterus and previous abdominal surgery are considered limitations of R-LESS hysterectomy and may require a longer learning curve [7].

The more recent development of a newer robotic system (da Vinci SP Surgical System) with articulating instruments and camera allow for intracorporeal triangulation and in March 2019 it was approved by the Food and Drug Administration (FDA) for urologic procedures and transoral otolaryngology. Although not currently FDA-approved for gynecologic surgery, it has already been successfully applied for a variety of gynecologic surgeries including hysterectomy [43].

The SP 1098 da Vinci SP Surgical System consists of the surgeon console, vision cart and patient cart, as with the previous da Vinci surgical platform. A single instrument arm is attached to the patient cart containing four instrument drives that control the articulating camera and up to three robotic instruments, which are inserted into the abdomen through a 25 mm SP multichannel port. The surgeon can control up to three 6 mm fully wristed, elbowed instruments. The instruments available at the moment are monopolar scissors, bipolar forceps, needle driver and forceps. The 10 mm oval endowrist SP camera has a 73° field of view and can be moved in a traditional fashion or in cobra mode preventing instrument collision and optimizing visualization.

The new single-port system enjoys several advantages such as increased dexterity and range of motion, camera mobility and intracorporeal instrument triangulation. Some alterations in technique may be required, for example, obtaining correct traction of the tissue is challenging due to the intrinsic limitation in lateral and anterior traction along the axis of the trocar.

## 6. Conclusions

Laparoendoscopic single site surgery (LESS) is a novel approach that has been associated with reduced postoperative pain, improved cosmetic outcome, avoidance of ancillary port complications and faster recovery compared to multiport laparoscopy. LESS should be considered an option of minimal invasive surgery, with specific instrumentation, learning curve and indications. LESS hysterectomy can be considered for selected groups of patients.

In recent years, the development of new technologies and instrumentations such as robotic assistance have been developed to overcome ergonomic complexity and to permit a diffusion of the single site approach.

Future developments and research in this field will broaden applications, diffusion and indications for LESS.

## Figures and Tables

**Table 1:** RCTs about hysterectomy for benign pathologies.

Author	Year	Sample Size SPL/MPL	Complications *n*, (%) SPL/MPL
Chen et al. [9]	2011	50/50	NS
Chung et al. [10]	2015	29/29	1/0
Jung et al. [11]	2011	30/34	(3.6/2)
Kim et al. [12]	2015	125/126	(8/10.3)
Li et al. [13]	2012	52/56	25/34 (*p* = 0.03)
Song et al. [14]	2013	20/19	0/0
Fanfani et al. [15]	2013	3/344	0/0
Song et al. [16]	2015	33/33	0/0

SPL: single port laparoscopy, MPL: multiport laparoscopy.

**Table 2:** Studies on R-LESS hysterectomy.

Author	Type of Study	No Patients	Indication	Postoperative
				Complication
Corrado et al. [22]	Prospective series	125 RSP	Oncology	10 (8%)
Jayakumaran et al. [23]	Retrospective series	35 RSP	Benign	-
Cela et al. [24]	Retrospective series	12RSP	Benign and Oncology	-
		15 RMP		
Gungor et al. [25]	Retrospective Case control	20 RSP	Benign	RSP 0
		25 LESS		RMP 0
Bogliolo et al. [26]	Retrospective Case control	45 RSP	Benign and oncology	RSP 1 (2.2%)
		59 RMP		RMP 2 (3.4%)
Paek et al. [27]	Retrospective	25 RSP	Benign	RSP 0
		100 LESS		LESS 2
Scheib et al. [28]	Prospective series	40 RSP		2.50%
Fagotti et al. [29]	Retrospective Case control	19RSP	Oncology	RSP 1 (5.9%)
		38 LESS		LESS 0
De Meritens [30]	Retrospective series	83RSP	Benign	2.4%
Akdemir et al. [31]	Retrospective Case control	24 RSP	Benign	-
		34 LESS		
Moukarze et al. [32]	Retrospective Case control	14RSP	Oncology	-
		13RMP		
Mereu et al. [33]	Prospective Case control	25 RSP	Oncology	RSP 1
		51 RMP		RMP 2
Hachem et al. [34]	Retrospective Case control	14 RSP	Benign and oncology	RSP 1
		43 LSC		LSC 0
Lopez et al. [35]	Retrospective series	50 RSP	Benign	RSP 1
		50 LESS		LESS 2
Yoo et al. [20]	Retrospective series	182RSS	Benign	-
Gupta et al. [36]	Retrospective series	49 RSP	Benign	RSP 2 (4.1%)
		36 RMP		RMP 0
		44 LSC		LSC 1 (2.3%)
Chen et al. [37]	Retrospective series	26 RSP	Benign	RSP 0
		57 RMP		RMP 0
Sendag et al. [38]	Retrospective series	24 RSP	Benign	-
Chung et al. [39]	Retrospective series	15 RSP	Oncoloy	RSP 1

RSP: robotic single port, RMP robotic multi-port, LESS: laparoendoscopic surgery, LSC: laparoscopy.

## Data Availability

Not applicable.
